# Telomere length and common disease: study design and analytical challenges

**DOI:** 10.1007/s00439-015-1563-4

**Published:** 2015-05-19

**Authors:** Jennifer H. Barrett, Mark M. Iles, Alison M. Dunning, Karen A. Pooley

**Affiliations:** Section of Epidemiology and Biostatistics, Leeds Institute of Cancer and Pathology, University of Leeds, St James’s University Hospital, Beckett Street, Leeds, LS9 7TF UK; Department of Oncology, Centre for Cancer Genetic Epidemiology, University of Cambridge, Strangeways Research Laboratory, Worts Causeway, Cambridge, CB1 8RN UK

## Abstract

Telomeres, the repetitive sequences that protect the ends of chromosomes, help to maintain genomic integrity and are of key importance to human health. The aim here is to give an overview of the evidence for the importance of telomere length (TL) to the risk of common disease, considering the strengths and weaknesses of different epidemiological study designs. Methods for measuring TL are described, all of which are subject to considerable measurement error. TL declines with age and varies in relation to factors such as smoking and obesity. It is also highly heritable (estimated heritability of ~40 to 50 %), and genome-wide studies have identified a number of associated genetic variants. Epidemiological studies have shown shorter TL to be associated with risk of a number of common diseases, including cardiovascular disease and some cancers. The relationship with cancer appears complex, in that longer telomeres are associated with higher risk of some cancers. Prospective studies of the relationship between TL and disease, where TL is measured before diagnosis, have numerous advantages over retrospective studies, since they avoid the problems of reverse causality and differences in sample handling, but they are still subject to potential confounding. Studies of the genetic predictors of TL in relation to disease risk avoid these drawbacks, although they are not without limitations. Telomere biology is of major importance to the risk of common disease, but the complexities of the relationship are only now beginning to be understood.

## Introduction

### Telomeres

Human chromosomes are capped and stabilised by telomeres. They are comprised of several thousand copies of a hexamer repeat sequence (TTAGGG)n, a single-stranded 3′ G-rich overhang, and a plethora of generic DNA-binding proteins, tankyrases, and specific telomere-binding proteins collectively termed the ‘shelterin’ complex (Baird 2006; Moyzis et al. 1988; Verdun and Karlseder 2007). Telomeres prevent chromosome ends from being recognised as damaged DNA in need of double-strand break repair and, as a result, protect against chromosome–chromosome fusions and rearrangements, helping maintain genomic integrity (Wright and Shay 2005; Murnane 2006; Blackburn 2001). The telomere has been likened to the plastic tip (aglet) at the end of a shoelace as it prevents degradation and ‘fraying’ of the lace or chromosome. Telomeres are built up in embryonic cells by telomerase, a ribonucleoprotein consisting of an oestrogen-responsive reverse transcriptase component (TERT) and an RNA subunit (TERC) (Greider and Blackburn 1996). They are heterogeneous in length, varying between chromosomes and individuals, and show remarkable sequence homology throughout higher organisms (Blasco 2007; Lansdorp et al. 1996; Londono-Vallejo 2004; Meyne et al. 1989).

Telomeres tend to shorten with each cell division, as this highly repetitive stretch of DNA is inefficiently copied. This is referred to as ‘the end replication problem’ (Valdes et al. 2005; Shay and Wright 2005; Levy et al. 1992); the leading 5′–3′ strand in the synthesis of new DNA can be successfully made to the end, but the lagging 3′–5′ strand cannot as its synthesis is more complex (Verdun and Karlseder 2006, 2007; Broccoli 2004; Petraccone et al. 2008). This leads to a progressive loss in mean telomere length (TL) of 15–66 bp per year (Valdes et al. 2005; Allsopp et al. 1992; Slagboom et al. 1994; Hastie et al. 1990; Mayer et al. 2006). The rate of telomere attrition is greatest in the first year of life [almost ten times the rate of loss compared to age 1–18 and 28 times the rate compared to age 19 and over (Aubert et al. 2012)], but the rate of attrition has also been shown to increase over the age of 50 years (Baird 2006; Cawthon et al. 2003).

Rare mutations in telomere maintenance genes, such as *TERT, RTEL1, DKC1* and *WRN*, can cause dramatically shortened telomeres and premature ageing and increase risk of several rare diseases, including dyskeratosis congenita, which is characterised by short telomeres and premature ageing (Gupta and Kumar 2010; Knight et al. 1999; Stuart et al. 2015; Crabbe et al. 2007). This contributes to the hypothesis of TL as a measure of ‘biological age’ of both the cell and the organism. The ‘Hayflick Limit’ of 52 mitoses, as measured in cell culture, is the approximate limit of replicative capacity for human cells (Weinstein and Ciszek 2002; Benetti et al. 2007; Hayflick 2003). Beyond this point, telomeres will be below a critical length and gross chromosomal rearrangements through repeated chromosomal breakage–fusion–bridge cycles will occur (Murnane 2006). In response to this ‘crisis’, as signalled via the telomere-associated proteins, the cell cycle will arrest (Wong et al. 2009; von Zglinicki 2003; Verdun et al. 2005). The vast majority of these arrested cells will undergo apoptosis or senesce, protecting the organism from creating a potentially tumourigenic cell (Wong et al. 2009; d’Adda di Fagagna et al. 2003). On rare occasions, a cell can escape apoptosis or senescence and completely bypass arrest. Approximately one in every ten million cells in ‘crisis’ can then re-lengthen their telomeres, to recap and protect whatever gross or localised DNA damage has occurred, through the derepression of telomerase (Stampfer and Yaswen 2003; Bodnar et al. 1998). More than 90 % of cancer cells show such renewed telomerase activity, indicating that this may play a key role in malignant transformation (Cawthon et al. 2003; Kolquist et al. 1998; Meeker 2006; Wu et al. 2003). It has therefore been hypothesised that shorter mean TL may predispose to a number of common diseases of ageing, including cardiovascular disease (Murnane 2006; van der Harst et al. 2010) and cancer (Weischer et al. 2013; Mirabello et al. 2010; Risques et al. 2007; Pooley et al. 2010), and thus could be used as a biomarker of disease risk.

### Measurement of telomere length

One of the major challenges has been the reliable measurement of TL to properly test such hypotheses. Blood leukocytes yield high-quality DNA that is suitable for TL assays, and blood is a convenient tissue to collect for epidemiological studies. Leukocyte TL is thus generally measured as a marker of overall TL, under the assumption that within an individual TL is generally strongly correlated across tissue types. After the isolation and characterisation of the telomere sequence in the late 1980s (Moyzis et al. 1988; Meyne et al. 1989), the first and only method of length determination for many years was TRF (terminal restriction fragment) Southern blotting (Bryant et al. 1997; Cherkas et al. 2008; Bataille et al. 2007). Although the method generated an absolute value (in kb) of mode TL for each sample, it was not particularly sensitive and used a large amount of DNA per sample (~1 µg).

In recent years, quantitative PCR (Q-PCR) assays to measure mean TL have been developed. Q-PCR assays can be used in high-throughput laboratories, since they are simple and rapid to perform and require minimal quantities of DNA (<100 ng per sample) (Cawthon 2002). They have been performed on tens of thousands of samples to date to study TL with respect to smoking (McGrath et al. 2007), obesity and dietary factors (Cassidy et al. 2010), stress (Surtees et al. 2011), general physical health (Harris et al. 2006), oxidative damage (Shen et al. 2009) and cancer risk (Pooley et al. 2010, 2013; Bojesen et al. 2013). The discovery of genetic variants that are significantly associated with Q-PCR-determined TL has provided proof that this is a valid and sensitive measurement tool (Codd et al. 2010; Pooley et al. 2013; Bojesen et al. 2013). However, debate over the relative merits of the different assays continues. In a recent evaluation, inter-laboratory coefficients of variation were higher for Q-PCR than for other methods (Martin-Ruiz et al. 2014). One disadvantage of Q-PCR is that absolute values are not generated, so it is difficult (but not impossible) to compare assay results from batch to batch, study to study and across different laboratories.

Other techniques used to investigate TL include (a). STELA: a PCR-based technology amplifying specific chromosomes (Xu and Blackburn 2007); (b). Q-FISH: quantitative fluorescence in situ hybridisation, whereby fluorescently labelled probes complimentary to the telomere repeats are hybridised to a metaphase spread of chromosomes (Zheng et al. 2009) and (c). Flow-FISH: an adaptation coupling Q-FISH incorporating flow cytometry, whereby the TL of multiple cell types can be measured and compared (Baerlocher et al. 2006). These other techniques, although incredibly specific and accurate, are very labour intensive and as yet unsuitable for large-scale studies of TL and disease. Most recently, the ‘TelSeq’ method has been shown to produce results that correlate well with existing technologies (Ding et al. 2014): whole-genome next-generation sequencing data are mined for reads that are rich in telomere sequence, and relative length is determined. With the potential to be relatively high-throughput, this may overtake Q-PCR as the method of choice in future studies.

## Telomere length

### Inter- and intra- individual variation in telomere length

There is considerable variation between individuals both in absolute TL and the rate of telomere shortening (Chen et al. 2011), even from birth (Okuda et al. 2002; Akkad et al. 2006). Much of this variation is attributable to either measurement error or variation in TL between cells in the same individual. Although consistent differences in both absolute TL and rate of attrition have been observed between different cell types, TLs have been found to be strongly correlated across different cell types within the same individual (Takubo et al. 2002; Aubert et al. 2012; Daniali et al. 2013).

### Demographic factors associated with telomere length

Various factors have been associated with inter-individual TL. Unsurprisingly, given the gradual erosion of telomeres with each cell division, age is by far the strongest predictor of individual TL, explaining an estimated 17.5 % of the inter-individual variation in TL (Daniali et al. 2013). Other demographic characteristics, such as sex and ethnicity, have been associated to a lesser extent. Men have significantly shorter telomeres than women, and their telomeres decline more rapidly with age (Aubert et al. 2012; Möller et al. 2009; Weischer et al. 2012; Mayer et al. 2006).

### Environmental factors associated with telomere length

Many environmental factors, particularly those indicating an “unhealthy lifestyle” (including obesity, smoking, lack of exercise and alcohol use) have been frequently, though somewhat inconsistently, associated with shorter telomeres (Cherkas et al. 2008; Cassidy et al. 2010; Weischer et al. 2012; Mirabello et al. 2009; Strandberg et al. 2011; Nawrot et al. 2004; LaRocca et al. 2010) and with the rate of telomere shortening (Chen et al. 2011). Their estimated effect on TL is much less than that of ageing; for example telomeres have been estimated to be 240 bp shorter in obese women and 5 bp shorter for every pack year smoked (Valdes et al. 2005). Given that many of these factors are known to lead to oxidative stress [e.g. obesity and smoking (Burke and Fitzgerald 2003; Dandona et al. 2004)] and that oxidative stress is known to accelerate shortening of telomeres (von Zglinicki 2000, 2002; Houben et al. 2008), they may well be directly affecting TL. However, given that both TL and these factors are changing and having an effect over the entire lifespan of an individual, it is difficult to establish directionality of effect, particularly when the various factors are so highly correlated. Evidence for the causality of a trait may be strengthened by showing that genetic predictors of that trait are also predictors of TL; for example, in a large study, a genetic predictor of body mass index was investigated, but not shown to be associated with TL (Du et al. 2013).

### Hereditary factors associated with telomere length

In addition to the rare genetic mutations discussed earlier, at least some of the population variation in TL is explained by common genetic polymorphisms. Before any genotyping was conducted, various studies had shown that TL was heritable, with a strong correlation (*r*^2^ = 0.25) between maternal and newborn TLs (Akkad et al. 2006); heritability estimates range from 36 to 86 % in twin and other familial studies (Slagboom et al. 1994; Vasa-Nicotera et al. 2005; Njajou et al. 2007; Bakaysa et al. 2007; Atzmon et al. 2009). It should be remembered that much of this heritability may be due to shared environment, which is notably not modelled in those studies that estimate heritability to be over 50 %. Family-based linkage analyses have also been conducted in two studies, finding significant evidence for linkage at 12q12.22 (Vasa-Nicotera et al. 2005; Mangino et al. 2008) and 14q23.2 (Andrew et al. 2006), although neither study appears to replicate the other’s findings.

The natural next step is to try to identify specific genetic regions that are associated with TL in a genome-wide association study (GWAS). The first GWAS to reach genome-wide significance identified a locus that includes *TERC* (which encodes the telomerase RNA component), with each copy of the minor allele conferring a roughly 75 bp reduction in TL (Codd et al. 2010). Subsequent GWAS have identified further single-nucleotide polymorphisms (SNPs) associated at genome-wide significance with TL including in the vicinity of *OBFC1*, which encodes the human homolog of a yeast protein involved in the replication and capping of telomeres (Levy et al. 2010), *CTC1*, which is also involved in telomere maintenance (Mangino et al. 2012), and *ZNF676*, a zinc finger protein whose role in telomere biology is unknown (Mangino et al. 2012). By far the biggest GWAS of TL was a meta-analysis including 37,684 individuals with replication in 10,739 individuals (Codd et al. 2013). This replicated the associations with *TERC* and *OBFC1*, revealed novel associations between TL and SNPs in the region of 3 genes known to be involved in telomere biology (*TERT*, *NAF1* and *RTEL1*) and found novel associations at two further loci (19p12 and 2p16.2) with no obvious candidate telomere-related genes. Despite the size of the study, the total variance in TL explained by the seven variants reaching genome-wide significance was only about 1 %, leaving the majority of the genetic variation influencing TL unexplained.

Pooley et al. (2013) also reported variants of genome-wide significance with TL in *TERC*, *TERT* and *OBFC1* in the analysis of a custom genotyping array (“iCOGS”) in breast cancer cases (*n* = 11,024) and healthy controls (*n* = 15,065). There was also supportive evidence (*p* < 5 × 10^−4^) of associations with SNPs in *NAF1*, *RTEL1* and at 2p16.2. However, at 2p16.2, they found the minor allele of surrogate SNP (rs10165485) to be associated with longer TL in contrast to the published rs11125529 association with shorter TL (Codd et al. 2010) (pairwise *r*^2^ between SNPs = 0.98). The reportedly associated SNP at 19p12 was not directly genotyped, nor was there any good surrogate, so this association could not be tested.

## Epidemiological evidence of the relationship between telomere length and common disease

Epidemiological studies have established an association between shorter TL and the risk of various age-related common diseases.

### Cardiovascular and metabolic disease

Haycock et al. (2014) recently reviewed the evidence for such an association with cardiovascular disease (coronary heart disease and cerebrovascular disease), conducting a meta-analysis of 24 studies. Evidence was seen of a relationship with coronary heart disease: estimated relative risk (RR) (comparing the shortest versus the longest third of TL) 1.54, 95 % confidence interval (1.30, 1.83), with moderate between-study heterogeneity (*I*^2^ = 64 %), and the association remained when adjusting for publication bias or restricting to prospective studies. A similar effect size was seen for the association with cerebrovascular disease [RR 1.42 (1.11, 1.81)], although there was no real evidence for an effect when restricting the meta-analysis to prospective studies.

In a similar meta-analysis, Zhao et al. (2013) concluded that shorter telomere length is associated with an increased risk of type 2 diabetes, although this meta-analysis did not distinguish between prospective and retrospective studies. In a more recently reported analysis (Willeit et al. 2014) based on the prospective Bruneck Study, TL was measured on three occasions, spanning 15 years, but only a small number of participants (44/606) developed type 2 diabetes. When correcting for regression dilution through analysis of the repeated TL measures, there was evidence of increased risk comparing the bottom to the top quarter of TL [RR 3.24 (1.29, 8.15)]. Upon meta-analysis with two other prospective studies, the estimated RR was 1.31 (1.07, 1.60), with moderate between-study heterogeneity (*I*^2^ = 69 %).

### Cancer

The relationship with cancer seems to be more complex. Wentzensen et al. (2011) and Ma et al. (2011) conducted similar meta-analyses of 25–29 epidemiological studies of TL and cancer risk published prior to 2010, including 13 different cancers and overall incident cancer. From a random effects meta-analysis of all studies, the RR of cancer to those with telomeres in the shortest quarter of TL compared with the longest was 1.96 (1.37, 2.81) (Wentzensen et al. 2011), but the between-study heterogeneity was substantial (*I*^2^ = 94 %). As the authors note, this may be due, at least in part, to differences between specific cancers. Strong evidence of association was seen between shorter telomeres and increased risk of bladder and gastric cancer, but no evidence of an association was seen with various other cancers, including breast cancer. Indeed there is accumulating evidence that, in contrast to the common pattern, longer telomeres are associated with increased risk of certain cancers, including melanoma (Nan et al. 2011; Burke et al. 2013), soft tissue sarcoma (Xie et al. 2013), B cell lymphoma (Hosnijeh et al. 2014) and lung cancer adenocarcinoma (Sanchez-Espiridion et al. 2014), suggesting perhaps that telomere maintenance inhibits apoptosis and increases the likelihood of malignancy in the development of these cancers. A number of recent studies have suggested there may be a non-monotonic relationship between TL and the risk of certain cancers, with higher risk at both extremes of TL (Qu et al. 2013; Skinner et al. 2012; Cui et al. 2012; Wang et al. 2014), but to date there has not been a sufficient number of large high-quality studies to confirm this pattern. Heterogeneity between studies may thus be due to combining related, but distinct, diseases that are influenced quite differently by TL.

### Retrospective and prospective studies

Heterogeneity may also arise through differences in study design and conduct. Even when there is emerging evidence of consistent association between TL and disease the reasons for the association remain uncertain.

Some of the meta-analyses have allowed comparisons to be made between retrospective studies (where TL is measured after disease diagnosis) and prospective studies (where TL may be measured a considerable time before diagnosis). Where estimates from these types of study are reported separately (e.g. Haycock et al. 2014; Wentzensen et al. 2011), the effect sizes tend to be larger from the retrospective studies, suggesting that reverse causality or other aspects of residual confounding contribute to the estimates. Pooley et al. (2010) observed far smaller estimates of risk from prospective compared with retrospective studies of TL and the risk of breast and colorectal cancer. Weischer et al. (2013) studied a prospective cohort study of over 47,000 individuals from Denmark, 3142 of whom received a cancer diagnosis during follow-up. Although the unadjusted hazard ratio for cancer risk was 1.74 (1.58, 1.93) for shortest versus longest quartile of TL, of similar magnitude to the meta-analyses (Wentzensen et al. 2011; Ma et al. 2011), the association disappeared after adjusting for other risk factors, primarily age. Reverse causality could be due to the disease process itself (for example increased levels of oxidative stress among cases) or to treatment effects.

Even in prospective studies, confounding by joint risk factors remains a strong possibility. Although in some studies, adjustment is made for potential confounders, residual confounding cannot be ruled out, especially as several common risk factors for these diseases are also related to TL, as outlined earlier.

### Genetic studies

To overcome concerns about both reverse causality and confounding, an increasing number of studies have examined the influence of telomere-related genes on disease risk, the rationale for which is considered more fully below. Polymorphisms in genes known to be involved in telomere maintenance have been investigated in genetic association studies of common disease. Most notably, polymorphisms in or around *TERT* (encoding telomerase reverse transcriptase) are strongly associated with various cancers, including breast, bladder and prostate cancers and melanoma (Rafnar et al. 2009; Bojesen et al. 2013).

However, the most strongly associated SNPs and even the direction of association differ between cancers. The effects may reflect differences in the direction of the associations with TL itself. For example *TERT* SNP alleles associated with longer TL in Bojesen et al. (2013) were also associated with increased risk of melanoma (Barrett et al. 2015), mirroring the observed association between longer TL and melanoma risk. In contrast, the minor allele of SNP rs2736108, associated with longer telomeres, is associated with lower risks for oestrogen receptor (ER)-negative (*p* = 10^−8^) and BRCA1 mutation carrier (*p* = 10^−5^) breast cancers (Bojesen et al. 2013). For hormonal cancers, the functional variants (rs10069690 and rs2242652) with the biggest effects on risk have no direct effects on TL, and, conversely, variants (rs7705526 and rs2736108), which are clearly drivers of TL, have smaller, secondary effects on breast and ovarian cancers (Bojesen et al. 2013; Terry et al. 2012; Pellatt et al. 2013). Thus, the *TERT* gene clearly has multiple, pleotropic effects; 5′ variants affect promoter activity and TL, whilst more 3′ variants, affecting RNA splicing and a TERT silencer element, have roles in hormonal cancer development but not via changes to TL.

Since the recent GWASs of telomere length, and in particular the meta-analysis by Codd et al. (2013), a more systematic approach has been possible combining the effects of all SNPs known to be associated with TL into a single polygenic score. Codd et al. (2013) showed modest evidence for an effect of a polygenic score based on the 7 genome-wide significant telomere-associated SNPs identified in their meta-analysis with coronary artery disease (*p* = 0.014, from a study of over 22,000 cases and 64,000 controls). Using similar approaches, effects have also been observed for various cancers, including bladder cancer (Chang et al. 2012). In particular, Iles et al. (2014) showed very strong evidence for an effect of the polygenic score from the same 7 SNPs (Codd et al. 2013) on the risk of melanoma (*p* < 10^−8^). Individuals with a polygenic score in the highest quartile were at almost 30 % increased risk of melanoma compared with those with a score in the lowest quartile.

## Study design

Although findings are sometimes inconsistent, there is clear and emerging evidence of association between TL and common disease, and of differential effects on different diseases. Many different study designs have been used, some measuring leukocyte TL directly and others considering TL-associated genotypes.

Studies measuring leukocyte telomeres directly exploit the strong correlation between TL in different tissues within an individual, although the evidence for this is not extensive and concerns remain about the stability of telomere measurements. TL varies considerably throughout a person’s lifetime, due to both ageing and specific environmental or host stimuli, and using current technology there is considerable measurement error. The timing and method of TL measurement are therefore both important factors. Telomeres measured after diagnosis may well be affected by treatment or by the disease process itself. Thus, prospective studies, where TL is measured well before disease onset, are necessary to ensure the association is not due to reverse causality.

Many disease-related factors, including diet, sun exposure and smoking, are themselves associated with TL. Thus, even in prospective studies, it is difficult to rule out confounding. In many of the prospective studies conducted to date, adjustment is made for known confounders, but residual confounding cannot be ruled out.

These considerations, along with the high heritability of TL and recent discovery of TL-related genetic variants, have led to an interest in studies where the relationship between genetic predictors of TL and disease risk is investigated. Clearly such studies avoid any possibility of reverse causality. Confounding is also unlikely, provided population stratification is properly accounted for. Is it therefore possible to invoke Mendelian Randomisation principles (Katan 1986; Davey Smith and Ebrahim 2003) and infer that an association between the genetic risk factors and disease demonstrates a causal role for TL in disease risk?

One of the assumptions behind Mendelian randomisation is that the genetic risk variants do not have a direct effect on disease risk other than through the putative causal factor (TL). However, some observations cast doubt on this assumption. Firstly, as described above in relation to *TERT*, genes can clearly have pleiotropic effects. It is also possible that specific genetic variants influence not only TL but also other aspects of telomere biology or DNA repair, and their association with disease risk may not be due to TL per se. A second observation relates to the relationship between genetic predictors of TL and risk of melanoma (Iles et al. 2014). The association is much stronger than would be expected if the risk were mediated through TL alone, given that the genetic predictors only explain about 1 % of the variation in measured TL (Codd et al. 2013). This may well be partly explained by measurement error and intra-individual variability of TL, but it could also be due to pleiotropic effects of the variants.

## Conclusions

The observed associations between TL and the risk of many different diseases suggest a role that is fundamental to health at the level of the cell and the organism. However, despite these strong associations, it is still unclear whether TL is itself causal or is a biomarker of underlying disease-related mechanisms. Interventions aimed at increasing TL for the purpose of halting or reversing the ageing process or preventing disease, are thus not supported by current evidence. Even as a biomarker of disease, TL does not currently have clinical utility in a population setting; associations with disease are complex, TL alone is not sufficiently predictive of risk and is subject to considerable measurement error. As understanding develops and evidence accumulates, TL may in the future provide a useful biomarker of risk or of disease progression.

Ideally, large prospective studies would be conducted with longitudinal measures of TL, extensive genotyping and detailed measures of phenotype and exposure to further understanding of the relationship with health. Since the measurement of TL is unstable, a key requirement is that sample collection and processing should be as uniform as possible. Such prospective studies would allow investigation of the relationship between genetic predictors of TL on disease risk, while adjusting for measured TL.

Further insights are also needed into telomere biology. It seems likely that the sole focus on TL, rather than other features of telomere maintenance and stability, is too simplistic. Similarly, the assumption that leukocyte TL is reflected in disease-relevant tissue needs further investigation, and consideration should be given to measuring TL at more than one time point. The observation that longer telomeres are associated with greater risk of some cancers also complicates the prevailing view that longer telomeres are always advantageous.

Large-scale prospective studies of disease that consistently and reliably measure TL are rare and expensive to conduct. However, large-scale datasets specifically designed to study the relationship between germline variation and disease risk have been established for most common disorders. The majority of these will not be suitable for the measurement of TL, given the sensitivity of TL measurement to sample handling and the variability of TL over time. Thus, at least until more reliable cost-effective methods of TL measurement become available, one of the most promising approaches to understanding the relationship between telomere features and disease is to study the genetic factors that underlie them.

## References

[CR1] Akkad A, Hastings R, Konje JC, Bell SC, Thurston H, Williams B (2006). Telomere length in small-for-gestational-age babies. BJOG.

[CR2] Allsopp RC, Vaziri H, Patterson C, Goldstein S, Younglai EV, Futcher AB, Greider CW, Harley CB (1992). Telomere length predicts replicative capacity of human fibroblasts. Proc Natl Acad Sci.

[CR3] Andrew T, Aviv A, Falchi M, Surdulescu GL, Gardner JP, Lu X, Kimura M, Kato BS, Valdes AM, Spector TD (2006). Mapping genetic loci that determine leukocyte telomere length in a large sample of unselected female sibling pairs. Am J Hum Genet.

[CR4] Atzmon G, Cho M, Cawthon RM, Budagov T, Katz M, Yang X, Siegel G, Bergman A, Huffman DM, Schechter CB, Wright WE, Shay JW, Barzilai N, Govindaraju DR, Suh Y (2009). Evolution in health and medicine Sackler colloquium: genetic variation in human telomerase is associated with telomere length in Ashkenazi centenarians. Proc Natl Acad Sci.

[CR5] Aubert G, Baerlocher GM, Vulto I, Poon SS, Lansdorp PM (2012). Collapse of telomere homeostasis in hematopoietic cells caused by heterozygous mutations in telomerase genes. PLoS Genet.

[CR6] Baerlocher GM, Vulto I, de Jong G, Lansdorp PM (2006). Flow cytometry and FISH to measure the average length of telomeres (flow FISH). Nat Protoc.

[CR7] Baird DM (2006). Telomeres. Exp Gerontol.

[CR8] Bakaysa SL, Mucci LA, Slagboom PE, Boomsma DI, McClearn GE, Johansson B, Pedersen NL (2007). Telomere length predicts survival independent of genetic influences. Aging Cell.

[CR9] Barrett JH, Taylor JC, Bright C, Harland M, Dunning AM, Akslen LA, Andresen PA, Avril MF, Azizi E, Bianchi Scarrà G, Brossard M, Brown KM, Dębniak T, Elder DE, Friedman E, Ghiorzo P, Gillanders EM, Gruis NA, Hansson J, Helsing P, Hočevar M, Höiom V, Ingvar C, Landi MT, Lang J, Lathrop GM, Lubiński J, Mackie RM, Molven A, Novaković S, Olsson H, Puig S, Puig-Butille JA, van der Stoep N, van Doorn R, van Workum W, Goldstein AM, Kanetsky PA, Pharoah PD, Demenais F, Hayward NK, Newton Bishop JA, Bishop DT, Iles MM, GenoMEL Consortium (2015) Fine mapping of genetic susceptibility loci for melanoma reveals a mixture of single variant and multiple variant regions. Int J Cancer 136:1351–1360 10.1002/ijc.29099 PMC4328144 25077817

[CR10] Bataille V, Kato BS, Falchi M, Gardner J, Kimura M, Lens M, Perks U, Valdes AM, Bennett DC, Aviv A, Spector TD (2007). Nevus size and number are associated with telomere length and represent potential markers of a decreased senescence in vivo. Cancer Epidemiol Biomark Prev.

[CR11] Benetti R, Garcia-Cao M, Blasco MA (2007). Telomere length regulates the epigenetic status of mammalian telomeres and subtelomeres. Nat Genet.

[CR12] Blackburn EH (2001). Switching and signaling at the telomere. Cell.

[CR13] Blasco MA (2007). Telomere length, stem cells and aging. Nat Chem Biol.

[CR14] Bodnar AG, Ouellette M, Frolkis M, Holt SE, Chiu CP, Morin GB, Harley CB, Shay JW, Lichtsteiner S, Wright WE (1998). Extension of life-span by introduction of telomerase into normal human cells. Science.

[CR15] Bojesen SE, Pooley KA, Johnatty SE, Beesley J, Michailidou K, Tyrer JP, Edwards SL, Pickett HA, Shen HC, Smart CE, Hillman KM, Mai PL, Lawrenson K, Stutz MD, Lu Y, Karevan R, Woods N, Johnston RL, French JD, Chen X, Weischer M, Nielsen SF, Maranian MJ, Ghoussaini M, Ahmed S, Baynes C, Bolla MK, Wang Q, Dennis J, McGuffog L, Barrowdale D, Lee A, Healey S, Lush M, Tessier DC, Vincent D, Bacot F, Australian Cancer Study, Australian Ovarian Cancer Study, Kathleen Cuningham Foundation Consortium for Research into Familial Breast Cancer (kConFab), Gene Environment Interaction and Breast Cancer (GENICA), Swedish Breast Cancer Study (SWE-BRCA), Hereditary Breast and Ovarian Cancer Research Group Netherlands (HEBON), Epidemiological study of BRCA1 & BRCA2 Mutation Carriers (EMBRACE), Genetic Modifiers of Cancer Risk in BRCA1/2 Mutation Carriers (GEMO), Vergote I, Lambrechts S, Despierre E, Risch HA, González-Neira A, Rossing MA, Pita G, Doherty JA, Alvarez N, Larson MC, Fridley BL, Schoof N, Chang-Claude J, Cicek MS, Peto J, Kalli KR, Broeks A, Armasu SM, Schmidt MK, Braaf LM, Winterhoff B, Nevanlinna H, Konecny GE, Lambrechts D, Rogmann L, Guénel P, Teoman A, Milne RL, Garcia JJ, Cox A, Shridhar V, Burwinkel B, Marme F, Hein R, Sawyer EJ, Haiman CA, Wang-Gohrke S, Andrulis IL, Moysich KB, Hopper JL, Odunsi K, Lindblom A, Giles GG, Brenner H, Simard J, Lurie G, Fasching PA, Carney ME, Radice P, Wilkens LR, Swerdlow A, Goodman MT, Brauch H, Garcia-Closas M, Hillemanns P, Winqvist R, Dürst M, Devilee P, Runnebaum I, Jakubowska A, Lubinski J, Mannermaa A, Butzow R, Bogdanova NV, Dörk T, Pelttari LM, Zheng W, Leminen A, Anton-Culver H, Bunker CH, Kristensen V, Ness RB, Muir K, Edwards R, Meindl A, Heitz F, Matsuo K, du Bois A, Wu AH, Harter P, Teo SH, Schwaab I, Shu XO, Blot W, Hosono S, Kang D, Nakanishi T, Hartman M, Yatabe Y, Hamann U, Karlan BY, Sangrajrang S, Kjaer SK, Gaborieau V, Jensen A, Eccles D, Høgdall E, Shen CY, Brown J, Woo YL, Shah M, Azmi MA, Luben R, Omar SZ, Czene K, Vierkant RA, Nordestgaard BG, Flyger H, Vachon C, Olson JE, Wang X, Levine DA, Rudolph A, Weber RP, Flesch-Janys D, Iversen E, Nickels S, Schildkraut JM, Silva Idos S, Cramer DW, Gibson L, Terry KL, Fletcher O, Vitonis AF, van der Schoot CE, Poole EM, Hogervorst FB, Tworoger SS, Liu J, Bandera EV, Li J, Olson SH, Humphreys K, Orlow I, Blomqvist C, Rodriguez-Rodriguez L, Aittomäki K, Salvesen HB, Muranen TA, Wik E, Brouwers B, Krakstad C, Wauters E, Halle MK, Wildiers H, Kiemeney LA, Mulot C, Aben KK, Laurent-Puig P, Altena AM, Truong T, Massuger LF, Benitez J, Pejovic T, Perez JI, Hoatlin M, Zamora MP, Cook LS, Balasubramanian SP, Kelemen LE, Schneeweiss A, Le ND, Sohn C, Brooks-Wilson A, Tomlinson I, Kerin MJ, Miller N, Cybulski C, Henderson BE, Menkiszak J, Schumacher F, Wentzensen N, Le Marchand L, Yang HP, Mulligan AM, Glendon G, Engelholm SA, Knight JA, Høgdall CK, Apicella C, Gore M, Tsimiklis H, Song H, Southey MC, Jager A, den Ouweland AM, Brown R, Martens JW, Flanagan JM, Kriege M, Paul J, Margolin S, Siddiqui N, Severi G, Whittemore AS, Baglietto L, McGuire V, Stegmaier C, Sieh W, Müller H, Arndt V, Labrèche F, Gao YT, Goldberg MS, Yang G, Dumont M, McLaughlin JR, Hartmann A, Ekici AB, Beckmann MW, Phelan CM, Lux MP, Permuth-Wey J, Peissel B, Sellers TA, Ficarazzi F, Barile M, Ziogas A, Ashworth A, Gentry-Maharaj A, Jones M, Ramus SJ, Orr N, Menon U, Pearce CL, Brüning T, Pike MC, Ko YD, Lissowska J, Figueroa J, Kupryjanczyk J, Chanock SJ, Dansonka-Mieszkowska A, Jukkola-Vuorinen A, Rzepecka IK, Pylkäs K, Bidzinski M, Kauppila S, Hollestelle A, Seynaeve C, Tollenaar RA, Durda K, Jaworska K, Hartikainen JM, Kosma VM, Kataja V, Antonenkova NN, Long J, Shrubsole M, Deming-Halverson S, Lophatananon A, Siriwanarangsan P, Stewart-Brown S, Ditsch N, Lichtner P, Schmutzler RK, Ito H, Iwata H, Tajima K, Tseng CC, Stram DO, van den Berg D, Yip CH, Ikram MK, Teh YC, Cai H, Lu W, Signorello LB, Cai Q, Noh DY, Yoo KY, Miao H, Iau PT, Teo YY, McKay J, Shapiro C, Ademuyiwa F, Fountzilas G, Hsiung CN, Yu JC, Hou MF, Healey CS, Luccarini C, Peock S, Stoppa-Lyonnet D, Peterlongo P, Rebbeck TR, Piedmonte M, Singer CF, Friedman E, Thomassen M, Offit K, Hansen TV, Neuhausen SL, Szabo CI, Blanco I, Garber J, Narod SA, Weitzel JN, Montagna M, Olah E, Godwin AK, Yannoukakos D, Goldgar DE, Caldes T, Imyanitov EN, Tihomirova L, Arun BK, Campbell I, Mensenkamp AR, van Asperen CJ, van Roozendaal KE, Meijers-Heijboer H, Collée JM, Oosterwijk JC, Hooning MJ, Rookus MA, van der Luijt RB, Os TA, Evans DG, Frost D, Fineberg E, Barwell J, Walker L, Kennedy MJ, Platte R, Davidson R, Ellis SD, Cole T, Bressac-de Paillerets B, Buecher B, Damiola F, Faivre L, Frenay M, Sinilnikova OM, Caron O, Giraud S, Mazoyer S, Bonadona V, Caux-Moncoutier V, Toloczko-Grabarek A, Gronwald J, Byrski T, Spurdle AB, Bonanni B, Zaffaroni D, Giannini G, Bernard L, Dolcetti R, Manoukian S, Arnold N, Engel C, Deissler H, Rhiem K, Niederacher D, Plendl H, Sutter C, Wappenschmidt B, Borg A, Melin B, Rantala J, Soller M, Nathanson KL, Domchek SM, Rodriguez GC, Salani R, Kaulich DG, Tea MK, Paluch SS, Laitman Y, Skytte AB, Kruse TA, Jensen UB, Robson M, Gerdes AM, Ejlertsen B, Foretova L, Savage SA, Lester J, Soucy P, Kuchenbaecker KB, Olswold C, Cunningham JM, Slager S, Pankratz VS, Dicks E, Lakhani SR, Couch FJ, Hall P, Monteiro AN, Gayther SA, Pharoah PD, Reddel RR, Goode EL, Greene MH, Easton DF, Berchuck A, Antoniou AC, Chenevix-Trench G, Dunning AM (2013) Multiple independent variants at the TERT locus are associated with telomere length and risks of breast and ovarian cancer. Nat Genet 45:371–84 10.1038/ng.2566 PMC3670748 23535731

[CR16] Broccoli D (2004). Function, replication and structure of the mammalian telomere. Cytotechnology.

[CR17] Bryant JE, Hutchings KG, Moyzis RK, Griffith JK (1997). Measurement of telomeric DNA content in human tissues. Biotechniques.

[CR18] Burke A, Fitzgerald GA (2003). Oxidative stress and smoking-induced vascular injury. Prog Cardiovasc Dis.

[CR19] Burke LS, Hyland PL, Pfeiffer RM, Prescott J, Wheeler W, Mirabello L, Savage SA, Burdette L, Yeager M, Chanock S, De Vivo I, Tucker MA, Goldstein AM, Yang XR (2013). Telomere length and the risk of cutaneous malignant melanoma in melanoma-prone families with and without CDKN2A mutations. PLoS One.

[CR20] Cassidy A, De Vivo I, Liu Y, Han J, Prescott J, Hunter DJ, Rimm EB (2010). Associations between diet, lifestyle factors, and telomere length in women. Am J Clin Nutr.

[CR21] Cawthon RM (2002). Telomere measurement by quantitative PCR. Nucleic Acids Res.

[CR22] Cawthon RM, Smith KR, O’Brien E, Sivatchenko A, Kerber RA (2003). Association between telomere length in blood and mortality in people aged 60 years or older. Lancet.

[CR23] Chang J, Dinney CP, Huang M, Wu X, Gu J (2012). Genetic variants in telomere-maintenance genes and bladder cancer risk. PLoS One.

[CR24] Chen W, Kimura M, Kim S, Cao X, Srinivasan SR, Berenson GS, Kark JD, Aviv A (2011). Longitudinal versus cross-sectional evaluations of leukocyte telomere length dynamics: age-dependent telomere shortening is the rule. J Gerontol A Biol Sci Med Sci.

[CR25] Cherkas LF, Hunkin JL, Kato BS, Richards JB, Gardner JP, Surdulescu GL, Kimura M, Lu X, Spector TD, Aviv A (2008). The association between physical activity in leisure time and leukocyte telomere length. Arch Intern Med.

[CR26] Codd V, Mangino M, van der Harst P, Braund PS, Kaiser M, Beveridge AJ, Rafelt S, Moore J, Nelson C, Soranzo N, Zhai G, Valdes AM, Blackburn H, Mateo Leach I, de Boer RA, Kimura M, Aviv A, Wellcome Trust Case Control Consortium, Goodall AH, Ouwehand W, van Veldhuisen DJ, van Gilst WH, Navis G, Burton PR, Tobin MD, Hall AS, Thompson JR, Spector T, Samani NJ (2010) Common variants near TERC are associated with mean telomere length. Nat Genet 42:197–199 10.1038/ng.532 PMC3773906 20139977

[CR27] Codd V, Nelson CP, Albrecht E, Mangino M, Deelen J, Buxton JL, Hottenga JJ, Fischer K, Esko T, Surakka I, Broer L, Nyholt DR, Mateo Leach I, Salo P, Hägg S, Matthews MK, Palmen J, Norata GD, O’Reilly PF, Saleheen D, Amin N, Balmforth AJ, Beekman M, de Boer RA, Böhringer S, Braund PS, Burton PR, de Craen AJ, Denniff M, Dong Y, Douroudis K, Dubinina E, Eriksson JG, Garlaschelli K, Guo D, Hartikainen AL, Henders AK, Houwing-Duistermaat JJ, Kananen L, Karssen LC, Kettunen J, Klopp N, Lagou V, van Leeuwen EM, Madden PA, Mägi R, Magnusson PK, Männistö S, McCarthy MI, Medland SE, Mihailov E, Montgomery GW, Oostra BA, Palotie A, Peters A, Pollard H, Pouta A, Prokopenko I, Ripatti S, Salomaa V, Suchiman HE, Valdes AM, Verweij N, Viñuela A, Wang X, Wichmann HE, Widen E, Willemsen G, Wright MJ, Xia K, Xiao X, van Veldhuisen DJ, Catapano AL, Tobin MD, Hall AS, Blakemore AI, van Gilst WH, Zhu H, Consortium C, Erdmann J, Reilly MP, Kathiresan S, Schunkert H, Talmud PJ, Pedersen NL, Perola M, Ouwehand W, Kaprio J, Martin NG, van Duijn CM, Hovatta I, Gieger C, Metspalu A, Boomsma DI, Jarvelin MR, Slagboom PE, Thompson JR, Spector TD, van der Harst P, Samani NJ (2013) Identification of seven loci affecting mean telomere length and their association with disease. Nat Genet 45:422–427 10.1038/ng.2528 PMC4006270 23535734

[CR28] Crabbe L, Jauch A, Naeger CM, Holtgreve-Grez H, Karlseder J (2007). Telomere dysfunction as a cause of genomic instability in Werner syndrome. Proc Natl Acad Sci.

[CR29] Cui Y, Cai Q, Qu S, Chow WH, Wen W, Xiang YB, Wu J, Rothman N, Yang G, Shu XO, Gao YT, Zheng W (2012). Association of leukocyte telomere length with colorectal cancer risk: nested case–control findings from the Shanghai Women’s Health Study. Cancer Epidemiol Biomark Prev.

[CR30] d’Adda di Fagagna F, Reaper PM, Clay-Farrace L, Fiegler H, Carr P, Von Zglinicki T, Saretzki G, Carter NP, Jackson SP (2003). A DNA damage checkpoint response in telomere-initiated senescence. Nature.

[CR31] Dandona P, Aljada A, Bandyopadhyay A (2004). Inflammation: the link between insulin resistance, obesity and diabetes. Trends Immunol.

[CR32] Daniali L, Benetos A, Susser E, Kark JD, Labat C, Kimura M, Desai K, Granick M, Aviv A (2013). Telomeres shorten at equivalent rates in somatic tissues of adults. Nat Commun.

[CR33] Davey Smith G, Ebrahim S (2003). Mendelian randomization: can genetic epidemiology contribute to understanding environmental determinants of disease?. Int J Epidemiol.

[CR34] Ding Z, Mangino M, Aviv A, Spector T, Durbin R, UK10K Consortium (2014). Estimating telomere length from whole genome sequence data. Nucleic Acids Res.

[CR35] Du M, Prescott J, Cornelis MC, Hankinson SE, Giovannucci E, Kraft P, De Vivo I (2013). Genetic predisposition to higher body mass index or type 2 diabetes and leukocyte telomere length in the Nurses’ Health Study. PLoS One.

[CR36] Greider CW, Blackburn EH (1996). Telomeres, telomerase and cancer. Sci Am.

[CR37] Gupta V, Kumar A (2010). Dyskeratosis congenita. Adv Exp Med Biol.

[CR38] Harris SE, Deary IJ, MacIntyre A, Lamb KJ, Radhakrishnan K, Starr JM, Whalley LJ, Shiels PG (2006). The association between telomere length, physical health, cognitive ageing, and mortality in non-demented older people. Neurosci Lett.

[CR39] Hastie ND, Dempster M, Dunlop MG, Thompson AM, Green DK, Allshire RC (1990). Telomere reduction in human colorectal carcinoma and with ageing. Nature.

[CR40] Haycock PC, Heydon EE, Kaptoge S, Butterworth AS, Thompson A, Willeit P (2014). Leucocyte telomere length and risk of cardiovascular disease: systematic review and meta-analysis. BMJ.

[CR41] Hayflick L (2003). Living forever and dying in the attempt. Exp Gerontol.

[CR42] Hosnijeh FS, Matullo G, Russo A, Guarrera S, Modica F, Nieters A, Overvad K, Guldberg P, Tjønneland A, Canzian F, Boeing H, Aleksandrova K, Trichopoulou A, Lagiou P, Trichopoulos D, Tagliabue G, Tumino R, Panico S, Palli D, Olsen KS, Weiderpass E, Dorronsoro M, Ardanaz E, Chirlaque MD, Sánchez MJ, Quirós JR, Venceslá A, Melin B, Johansson AS, Nilsson P, Borgquist S, Peeters PH, Onland-Moret NC, Bueno-de-Mesquita HB, Travis RC, Khaw KT, Wareham N, Brennan P, Ferrari P, Gunter MJ, Vineis P, Vermeulen R (2014). Prediagnostic telomere length and risk of B-cell lymphoma—results from the EPIC cohort study. Int J Cancer.

[CR43] Houben JM, Moonen HJ, van Schooten FJ, Hageman GJ (2008). Telomere length assessment: biomarker of chronic oxidative stress?. Free Radic Biol Med.

[CR44] Iles MM, Bishop DT, Taylor JC, Hayward NK, Brossard M, Cust AE, Dunning AM, Lee JE, Moses EK, Akslen LA; AMFS Investigators, Andresen PA, Avril MF, Azizi E, Scarrà GB, Brown KM, Dębniak T, Elder DE, Friedman E, Ghiorzo P, Gillanders EM, Goldstein AM, Gruis NA, Hansson J, Harland M, Helsing P, Hočevar M, Höiom V; IBD investigators, Ingvar C, Kanetsky PA, Landi MT, Lang J, Lathrop GM, Lubiński J, Mackie RM, Martin NG, Molven A, Montgomery GW, Novaković S, Olsson H, Puig S, Puig-Butille JA; QMEGA and QTWIN Investigators, Radford-Smith GL, Randerson-Moor J; SDH Study Group, van der Stoep N, van Doorn R, Whiteman DC, MacGregor S, Pooley KA, Ward SV, Mann GJ, Amos CI, Pharoah PD, Demenais F, Law MH, Newton Bishop JA, Barrett JH; GenoMEL Consortium (2014) The effect on melanoma risk of genes previously associated with telomere length. J Natl Cancer Inst 106(10). doi:10.1093/jnci/dju267 10.1093/jnci/dju267 PMC4196080 25231748

[CR45] Katan MB (1986). Apolipoprotein E isoforms, serum cholesterol, and cancer. Lancet.

[CR46] Knight SW, Heiss NS, Vulliamy TJ, Greschner S, Stavrides G, Pai GS, Lestringant G, Varma N, Mason PJ, Dokal I, Poustka A (1999). X-linked dyskeratosis congenita is predominantly caused by missense mutations in the DKC1 gene. Am J Hum Genet.

[CR47] Kolquist KA, Ellisen LW, Counter CM, Meyerson M, Tan LK, Weinberg RA, Haber DA, Gerald WL (1998). Expression of TERT in early premalignant lesions and a subset of cells in normal tissues. Nat Genet.

[CR48] Lansdorp PM, Verwoerd NP, van de Rijke FM, Dragowska V, Little MT, Dirks RW, Raap AK, Tanke HJ (1996). Heterogeneity in telomere length of human chromosomes. Hum Mol Genet.

[CR49] LaRocca TJ, Seals DR, Pierce GL (2010). Leukocyte telomere length is preserved with aging in endurance exercise-trained adults and related to maximal aerobic capacity. Mech Ageing Dev.

[CR50] Levy MZ, Allsopp RC, Futcher AB, Greider CW, Harley CB (1992). Telomere end-replication problem and cell aging. J Mol Biol.

[CR51] Levy D, Neuhausen SL, Hunt SC, Kimura M, Hwang SJ, Chen W, Bis JC, Fitzpatrick AL, Smith E, Johnson AD, Gardner JP, Srinivasan SR, Schork N, Rotter JI, Herbig U, Psaty BM, Sastrasinh M, Murray SS, Vasan RS, Province MA, Glazer NL, Lu X, Cao X, Kronmal R, Mangino M, Soranzo N, Spector TD, Berenson GS, Aviv A (2010). Genome-wide association identifies OBFC1 as a locus involved in human leukocyte telomere biology. Proc Natl Acad Sci.

[CR52] Londono-Vallejo JA (2004). Telomere length heterogeneity and chromosome instability. Cancer Lett.

[CR53] Ma H, Zhou Z, Wei S, Liu Z, Pooley KA, Dunning AM, Svenson U, Roos G, Hosgood HD, Shen M, Wei Q (2011). Shortened telomere length is associated with increased risk of cancer: a meta-analysis. PLoS One.

[CR54] Mangino M, Brouilette S, Braund P, Tirmizi N, Vasa-Nicotera M, Thompson JR, Samani NJ (2008). A regulatory SNP of the BICD1 gene contributes to telomere length variation in humans. Hum Mol Genet.

[CR55] Mangino M, Hwang SJ, Spector TD, Hunt SC, Kimura M, Fitzpatrick AL, Christiansen L, Petersen I, Elbers CC, Harris T, Chen W, Srinivasan SR, Kark JD, Benetos A, El Shamieh S, Visvikis-Siest S, Christensen K, Berenson GS, Valdes AM, Viñuela A, Garcia M, Arnett DK, Broeckel U, Province MA, Pankow JS, Kammerer C, Liu Y, Nalls M, Tishkoff S, Thomas F, Ziv E, Psaty BM, Bis JC, Rotter JI, Taylor KD, Smith E, Schork NJ, Levy D, Aviv A (2012). Genome-wide meta-analysis points to CTC1 and ZNF676 as genes regulating telomere homeostasis in humans. Hum Mol Genet.

[CR56] Martin-Ruiz CM, Baird D, Roger L, Boukamp P, Krunic D, Cawthon R, Dokter MM, van der Harst P, Bekaert S, de Meyer T, Roos G, Svenson U, Codd V, Samani NJ, McGlynn L, Shiels PG, Pooley KA, Dunning AM, Cooper R, Wong A, Kingston A, von Zglinicki T (2014) Reproducibility of telomere length assessment: an international collaborative study. Int J Epidemiol. pii: dyu191. [Epub ahead of print] 10.1093/ije/dyu191 PMC4681105 25239152

[CR57] Mayer S, Brüderlein S, Perner S, Waibel I, Holdenried A, Ciloglu N, Hasel C, Mattfeldt T, Nielsen KV, Möller P (2006). Sex-specific telomere length profiles and age-dependent erosion dynamics of individual chromosome arms in humans. Cytogenet Genome Res.

[CR58] McGrath M, Wong JY, Michaud D, Hunter DJ, De Vivo I (2007). Telomere length, cigarette smoking, and bladder cancer risk in men and women. Cancer Epidemiol Biomark Prev.

[CR59] Meeker AK (2006). Telomeres and telomerase in prostatic intraepithelial neoplasia and prostate cancer biology. Urol Oncol.

[CR60] Meyne J, Ratliff RL, Moyzis RK (1989). Conservation of the human telomere sequence (TTAGGG)n among vertebrates. Proc Natl Acad Sci.

[CR61] Mirabello L, Huang WY, Wong JY, Chatterjee N, Reding D, Crawford ED, De Vivo I, Hayes RB, Savage SA (2009). The association between leukocyte telomere length and cigarette smoking, dietary and physical variables, and risk of prostate cancer. Aging Cell.

[CR62] Mirabello L, Garcia-Closas M, Cawthon R, Lissowska J, Brinton LA, Pepłońska B, Sherman ME, Savage SA (2010). Leukocyte telomere length in a population-based case–control study of ovarian cancer: a pilot study. Cancer Causes Control.

[CR63] Möller P, Mayer S, Mattfeldt T, Müller K, Wiegand P, Brüderlein S (2009). Sex-related differences in length and erosion dynamics of human telomeres favor females. Aging (Albany NY).

[CR64] Moyzis RK, Buckingham JM, Cram LS, Dani M, Deaven LL, Jones MD, Meyne J, Ratliff RL, Wu JR (1988). A highly conserved repetitive DNA sequence, (TTAGGG)n, present at the telomeres of human chromosomes. Proc Natl Acad Sci.

[CR65] Murnane JP (2006). Telomeres and chromosome instability. DNA Repair (Amst).

[CR66] Nan H, Du M, De Vivo I, Manson JE, Liu S, McTiernan A, Curb JD, Lessin LS, Bonner MR, Guo Q, Qureshi AA, Hunter DJ, Han J (2011). Shorter telomeres associate with a reduced risk of melanoma development. Cancer Res.

[CR67] Nawrot TS, Staessen JA, Gardner JP, Aviv A (2004). Telomere length and possible link to X chromosome. Lancet.

[CR68] Njajou OT, Cawthon RM, Damcott CM, Wu SH, Ott S, Garant MJ, Blackburn EH, Mitchell BD, Shuldiner AR, Hsueh WC (2007). Telomere length is paternally inherited and is associated with parental lifespan. Proc Natl Acad Sci.

[CR69] Okuda K, Bardeguez A, Gardner JP, Rodriguez P, Ganesh V, Kimura M, Skurnick J, Awad G, Aviv A (2002). Telomere length in the newborn. Pediatr Res.

[CR70] Pellatt AJ, Wolff RK, Torres-Mejia G, John EM, Herrick JS, Lundgreen A, Baumgartner KB, Giuliano AR, Hines LM, Fejerman L, Cawthon R, Slattery ML (2013). Telomere length, telomere-related genes, and breast cancer risk: the Breast Cancer Health Disparities Study. Genes Chromosomes Cancer.

[CR71] Petraccone L, Trent JO, Chaires JB (2008). The tail of the telomere. J Am Chem Soc.

[CR72] Pooley KA, Sandhu MS, Tyrer J, Shah M, Driver KE, Luben RN, Bingham SA, Ponder BA, Pharoah PD, Khaw KT, Easton DF, Dunning AM (2010). Telomere length in prospective and retrospective cancer case–control studies. Cancer Res.

[CR73] Pooley KA, Bojesen SE, Weischer M, Nielsen SF, Thompson D, Amin Al Olama A, Michailidou K, Tyrer JP, Benlloch S, Brown J, Audley T, Luben R, Khaw KT, Neal DE, Hamdy FC, Donovan JL, Kote-Jarai Z, Baynes C, Shah M, Bolla MK, Wang Q, Dennis J, Dicks E, Yang R, Rudolph A, Schildkraut J, Chang-Claude J, Burwinkel B, Chenevix-Trench G, Pharoah PD, Berchuck A, Eeles RA, Easton DF, Dunning AM, Nordestgaard BG (2013). A genome-wide association scan (GWAS) for mean telomere length within the COGS project: identified loci show little association with hormone-related cancer risk. Hum Mol Genet.

[CR74] Qu S, Wen W, Shu XO, Chow WH, Xiang YB, Wu J, Ji BT, Rothman N, Yang G, Cai Q, Gao YT, Zheng W (2013). Association of leukocyte telomere length with breast cancer risk: nested case–control findings from the Shanghai Women’s Health Study. Am J Epidemiol.

[CR75] Rafnar T, Sulem P, Stacey SN, Geller F, Gudmundsson J, Sigurdsson A, Jakobsdottir M, Helgadottir H, Thorlacius S, Aben KK, Blöndal T, Thorgeirsson TE, Thorleifsson G, Kristjansson K, Thorisdottir K, Ragnarsson R, Sigurgeirsson B, Skuladottir H, Gudbjartsson T, Isaksson HJ, Einarsson GV, Benediktsdottir KR, Agnarsson BA, Olafsson K, Salvarsdottir A, Bjarnason H, Asgeirsdottir M, Kristinsson KT, Matthiasdottir S, Sveinsdottir SG, Polidoro S, Höiom V, Botella-Estrada R, Hemminki K, Rudnai P, Bishop DT, Campagna M, Kellen E, Zeegers MP, de Verdier P, Ferrer A, Isla D, Vidal MJ, Andres R, Saez B, Juberias P, Banzo J, Navarrete S, Tres A, Kan D, Lindblom A, Gurzau E, Koppova K, de Vegt F, Schalken JA, van der Heijden HF, Smit HJ, Termeer RA, Oosterwijk E, van Hooij O, Nagore E, Porru S, Steineck G, Hansson J, Buntinx F, Catalona WJ, Matullo G, Vineis P, Kiltie AE, Mayordomo JI, Kumar R, Kiemeney LA, Frigge ML, Jonsson T, Saemundsson H, Barkardottir RB, Jonsson E, Jonsson S, Olafsson JH, Gulcher JR, Masson G, Gudbjartsson DF, Kong A, Thorsteinsdottir U, Stefansson K (2009). Sequence variants at the *TERT*-*CLPTM1L* locus associate with many cancer types. Nat Genet.

[CR76] Risques RA, Vaughan TL, Li X, Odze RD, Blount PL, Ayub K, Gallaher JL, Reid BJ, Rabinovitch PS (2007). Leukocyte telomere length predicts cancer risk in Barrett’s esophagus. Cancer Epidemiol Biomark Prev.

[CR77] Sanchez-Espiridion B, Chen M, Chang JY, Lu C, Chang DW, Roth JA, Wu X, Gu J (2014). Telomere length in peripheral blood leukocytes and lung cancer risk: a large case–control study in Caucasians. Cancer Res.

[CR78] Shay JW, Wright WE (2005). Senescence and immortalization: role of telomeres and telomerase. Carcinogenesis.

[CR79] Shen J, Gammon MD, Terry MB, Wang Q, Bradshaw P, Teitelbaum SL, Neugut AI, Santella RM (2009). Telomere length, oxidative damage, antioxidants and breast cancer risk. Int J Cancer.

[CR80] Skinner HG, Gangnon RE, Litzelman K, Johnson RA, Chari ST, Petersen GM, Boardman LA (2012). Telomere length and pancreatic cancer: a case–control study. Cancer Epidemiol Biomark Prev.

[CR81] Slagboom PE, Droog S, Boomsma DI (1994). Genetic determination of telomere size in humans: a twin study of three age groups. Am J Hum Genet.

[CR82] Stampfer MR, Yaswen P (2003). Human epithelial cell immortalization as a step in carcinogenesis. Cancer Lett.

[CR83] Strandberg TE, Saijonmaa O, Tilvis RS, Pitkälä KH, Strandberg AY, Miettinen TA, Fyhrquist F (2011). Association of telomere length in older men with mortality and midlife body mass index and smoking. J Gerontol A Biol Sci Med Sci.

[CR84] Stuart BD, Choi J, Zaidi S, Xing C, Holohan B, Chen R, Choi M, Dharwadkar P, Torres F, Girod CE, Weissler J, Fitzgerald J, Kershaw C, Klesney-Tait J, Mageto Y, Shay JW, Ji W, Bilguvar K, Mane S, Lifton RP, Garcia CK (2015). Exome sequencing links mutations in PARN and RTEL1 with familial pulmonary fibrosis and telomere shortening. Nat Gen.

[CR85] Surtees PG, Wainwright NW, Pooley KA, Luben RN, Khaw KT, Easton DF, Dunning AM (2011). Life stress, emotional health, and mean telomere length in the European Prospective Investigation into Cancer (EPIC)-Norfolk population study. J Gerontol A Biol Sci Med Sci.

[CR86] Takubo K, Izumiyama-Shimomura N, Honma N, Sawabe M, Arai T, Kato M, Oshimura M, Nakamura K (2002). Telomere lengths are characteristic in each human individual. Exp Gerontol.

[CR87] Terry KL, Tworoger SS, Vitonis AF, Wong J, Titus-Ernstoff L, De Vivo I, Cramer DW (2012). Telomere length and genetic variation in telomere maintenance genes in relation to ovarian cancer risk. Cancer Epidemiol Biomark Prev.

[CR88] Valdes AM, Andrew T, Gardner JP, Kimura M, Oelsner E, Cherkas LF, Aviv A, Spector TD (2005). Obesity, cigarette smoking, and telomere length in women. Lancet.

[CR89] van der Harst P, de Boer RA, Samani NJ, Wong LS, Huzen J, Codd V, Hillege HL, Voors AA, van Gilst WH, Jaarsma T, van Veldhuisen DJ (2010). Telomere length and outcome in heart failure. Ann Med.

[CR90] Vasa-Nicotera M, Brouilette S, Mangino M, Thompson JR, Braund P, Clemitson JR, Mason A, Bodycote CL, Raleigh SM, Louis E, Samani NJ (2005). Mapping of a major locus that determines telomere length in humans. Am J Hum Genet.

[CR91] Verdun RE, Karlseder J (2006). The DNA damage machinery and homologous recombination pathway act consecutively to protect human telomeres. Cell.

[CR92] Verdun RE, Karlseder J (2007). Replication and protection of telomeres. Nature.

[CR93] Verdun RE, Crabbe L, Haggblom C, Karlseder J (2005). Functional human telomeres are recognized as DNA damage in G2 of the cell cycle. Mol Cell.

[CR94] von Zglinicki T (2000). Role of oxidative stress in telomere length regulation and replicative senescence. Ann NY Acad Sci.

[CR95] von Zglinicki T (2002). Oxidative stress shortens telomeres. Trends Biochem Sci.

[CR96] von Zglinicki T (2003). Telomeres, telomerase and the cancer cell: an introduction. Cancer Lett.

[CR97] Wang S, Chen Y, Qu F, He S, Huang X, Jiang H, Jin T, Wan S, Xing J (2014). Association between leukocyte telomere length and glioma risk: a case–control study. Neuro Oncol.

[CR98] Weinstein BS, Ciszek D (2002). The reserve-capacity hypothesis: evolutionary origins and modern implications of the trade-off between tumor-suppression and tissue repair. Exp Gerontol.

[CR99] Weischer M, Bojesen SE, Cawthon RM, Freiberg JJ, Tybjærg-Hansen A, Nordestgaard BG (2012). Short telomere length, myocardial infarction, ischemic heart disease, and early death. Arterioscler Thromb Vasc Biol.

[CR100] Weischer M, Bojesen SE, Cawthon RM, Freiberg JL, Tybærg-Hansen A, Nordestgaard BG (2013). Short telomere length, cancer survival, and cancer risk in 47,102 individuals. J Natl Cancer Inst.

[CR101] Wentzensen IM, Mirabello L, Pfeiffer RM, Savage SA (2011). The association of telomere length and cancer: a meta-analysis. Cancer Epidemiol Biomark Prev.

[CR102] Willeit P, Raschenberger J, Heydon EE, Tsimikas S, Haun M, Mayr A, Weger S, Witztum JL, Butterworth AS, Willeit J, Kronenberg F, Kiechl S (2014). Leucocyte telomere length and risk of type 2 diabetes mellitus: new prospective cohort study and literature-based meta-analysis. PLoS One.

[CR103] Wong LS, Oeseburg H, de Boer RA, van Gilst WH, van Veldhuisen DJ, van der Harst P (2009). Telomere biology in cardiovascular disease: the TERC-/- mouse as a model for heart failure and ageing. Cardiovasc Res.

[CR104] Wright WE, Shay JW (2005). Telomere-binding factors and general DNA repair. Nat Genet.

[CR105] Wu X, Amos CI, Zhu Y, Zhao H, Grossman BH, Shay JW, Luo S, Hong WK, Spitz MR (2003). Telomere dysfunction: a potential cancer predisposition factor. J Natl Cancer Inst.

[CR106] Xie H, Wu X, Wang S, Chang D, Pollock RE, Lev D, Gu J (2013). Long telomeres in peripheral blood leucocytes are associated with an increased risk of soft tissue sarcoma. Cancer.

[CR107] Xu L, Blackburn EH (2007). Human cancer cells harbor T-stumps, a distinct class of extremely short telomeres. Mol Cell.

[CR108] Zhao J, Miao K, Wang H, Ding H, Wang DW (2013). Association between telomere length and type 2 diabetes mellitus: a meta-analysis. PLoS One.

[CR109] Zheng YL, Hu N, Sun Q, Wang C, Taylor PR (2009). Telomere attrition in cancer cells and telomere length in tumor stroma cells predict chromosome instability in esophageal squamous cell carcinoma: a genome-wide analysis. Cancer Res.

